# Genomic and epidemiological insights into a non-toxigenic *Vibrio cholerae* O1 Ogawa from an autochthonous case in Brazil

**DOI:** 10.1007/s10096-026-05495-4

**Published:** 2026-04-15

**Authors:** André Felipe Santos, Pedro Panzenhagen, Verônica Dias Gonçalves, Laura Fabiana Vieira do Amparo, Joyce Figueira Pereira de Souza, Maria Eduarda Moreira, Maria Luiza Paula Pereira, Emily Moraes Roges, Lucia Helena Berto, Dália dos Rodrigues

**Affiliations:** 1 Oswaldo Cruz Institute (IOC), Oswaldo Cruz Foundation, Campus Maré, Rio de Janeiro, RJ 21040-361 Brazil; 2 Institute of Chemistry (IQ), Federal University of Rio de Janeiro (UFRJ), Cidade Universitária, Rio de Janeiro, RJ 21941-909 Brazil; 3 Ministry of Health, Brasilia, Brazil

**Keywords:** *Vibrio cholerae*, Genomic surveillance, Brazil, Non-toxigenic, Phylogenomics

## Abstract

*Vibrio cholerae* remains a global health threat, driven by epidemic strains and sporadic infections associated with non-toxigenic lineages. We report the genomic characterization of a *V. cholerae* O1 Ogawa isolate (V86/24) recovered from a human case in Brazil (2024) with no travel history. We performed antimicrobial susceptibility testing, whole-genome sequencing (WGS), and in silico analyses, including SNP-based phylogenetic and pangenome analyses (V86/24 + 62 public genomes; total *N* = 63), resistome, and virulome. The isolate showed intermediate susceptibility to imipenem but was susceptible to other tested antimicrobials. WGS identified V86/24 as sequence type ST170, lineage L3b.2, sublineage MX-2. It harbors pathogenicity islands VPI-1 and VPI-2 but lacks seventh-pandemic-associated islands (VSP-1, VSP-2) and classical CTXΦ-associated toxin genes (*ctxA*, *ctxB*, *ace*, *zot*). Pangenome analysis revealed 4,485 gene clusters; V86/24 carried eight unique clusters primarily mapped to a prophage-like region. Resistome profiling identified the intrinsic *varG* metallo-β-lactamase gene. The virulome retained alternative virulence and colonization factors, including TCP/ACF, RTX/MARTX, and an extensive type VI secretion system. Phylogenetically, V86/24 clusters with ST170 isolates circulating in the Americas, distinctly separated from seventh-pandemic lineages. Non-toxigenic *V. cholerae* O1 lineages can cause human infection. These findings underscore the critical need for integrated genomic surveillance, including pangenome profiling, beyond classical toxigenicity markers to detect emerging risks prior to epidemic spread.

**Supplementary Information:**

The online version contains supplementary material available at 10.1007/s10096-026-05495-4.

## Introduction

Infections caused by *Vibrio cholerae* remain a relevant public health concern, not only due to epidemic cholera driven by toxigenic O1 and O139 lineages, but also because of sporadic gastroenteritis cases associated with O1 and non-O1/non-O139 isolates outside endemic settings [1]. These events may arise from environmental exposure, silent local circulation, or unrecognized introduction, particularly when clinical presentation is not immediately attributed to *Vibrio* spp.

Classical cholera pathogenesis is tightly linked to acquisition and expression of the CTXΦ bacteriophage, encoding cholera toxin subunits (*ctx*A/*ctx*B), as well as accessory toxins (*ace* and *zot*), in combination with efficient intestinal colonization mediated by the toxin-coregulated pilus (TCP) [2]. These features are signatures of seventh pandemic (7PET) lineages and largely explain their epidemic potential [3]. However, increasing genomic and epidemiological evidence demonstrates that *ctx*AB-negative isolates can still cause clinically relevant disease, supported by alternative virulence repertoires rather than classical toxigenicity [4, 5].

Such alternative pathogenic profiles frequently include hemolysin (*hly*A), multifunctional auto processing repeats-in-toxin (RTX/MARTX) clusters, adhesion and colonization factors (e.g., accessory colonization factors *acf*A–*acf*D and *omp*U), quorum sensing systems, and secretion systems, particularly the type VI secretion system (T6SS) [1, 4]. Together, these determinants enhance intestinal fitness, bacterial competition, and environmental persistence, allowing non-toxigenic lineages to remain epidemiologically relevant despite lacking the CTXΦ module [4].

From an evolutionary and surveillance perspective, genomic islands play a key role in contextualizing *V. cholerae* diversity. While VSP-I and VSP-II are often associated with 7PET lineages and pandemic spread, VPI-1 and VPI-2 contribute primarily to colonization capacity, metabolic flexibility, and host interaction [6]. Distinct combinations of these islands have been described in non-pandemic lineages, reinforcing the importance of genome-based approaches to accurately interpret risk beyond serogroup or toxin gene presence alone [6].

Previous genomic studies have highlighted that the epidemiological relevance of Vibrio cholerae O1 cannot be interpreted solely on the basis of classical toxigenicity markers. Comparative genomics has shown that lateral gene transfer, recombination, and differential acquisition of mobile genetic elements contribute substantially to the emergence and diversification of pathogenic V. cholerae lineages. In addition, non-toxigenic environmental O1 strains from Haiti, including 2012Env-9 and 2012Env-390, demonstrated that ctxAB-negative O1 backgrounds may persist in aquatic environments while retaining evolutionary and public health relevance. These findings reinforce the importance of situating clinical non-toxigenic O1 isolates within a broader comparative genomic framework [7, 8].

Beyond the distinction between pandemic and non-pandemic O1 strains, recent population-genomic studies have shown that non-toxigenic O1 isolates of clinical relevance are not randomly distributed across the species, but often cluster within specific evolutionary backgrounds. In particular, ctxAB-negative, tcpA-positive O1 strains have been associated mainly with the broader L3b and L9 lineages, with L3b, and specifically its MX-2 sublineage, showing evidence of long-term persistence, regional circulation, and representation in Latin American datasets [9].

In parallel, antimicrobial resistance in *V. cholerae* has gained increasing attention. Although the species is generally susceptible to first-line agents, intrinsic and acquired mechanisms such as β-lactamases, envelope-associated modifications, and regulatory variants may subtly modulate susceptibility profiles, including to β-lactams and carbapenems [10, 11]. These features emphasize the need for integrated phenotypic and genomic assessment, particularly in clinical isolates where resistance may not be apparent [12].

In this context, the detection in 2024 of a human *V. cholerae* O1 Ogawa isolate in Brazil, without reported travel to endemic areas, is epidemiologically significant. This finding raises questions regarding local environmental exposure, undetected circulation of non-pandemic lineages, and the potential role of such strains as sentinels of future risk [1, 13]. Accordingly, this study aimed to comprehensively characterize the isolate V86/24 by integrating phenotypic antimicrobial susceptibility testing with whole-genome sequencing–based analyses, including resistome profiling, virulome characterization, and SNP-based phylogenetic positioning relative to publicly available genomes. By situating this isolate within a strategic evolutionary and epidemiological framework, we sought to clarify its relationship to pandemic and non-pandemic lineages and to discuss implications for clinical interpretation and genomic surveillance of *V. cholerae* in non-endemic settings.

## Materials and methods

### Isolate and antimicrobial susceptibility testing

Isolate V86/24 was recovered in 2024 from a 60-year-old male patient in Brazil presenting with diarrhea and a clinical background of achlorhydria, with no reported history of travel to cholera-endemic areas, and was identified as *Vibrio cholerae* O1 Ogawa. Antimicrobial susceptibility testing was performed by disk diffusion according to Clinical and Laboratory Standards Institute (CLSI) guidelines, criteria for *Vibrio* spp. when available; when no species-specific breakpoints were available, interpretation followed the CLSI guidance for infrequently isolated organisms. The antimicrobial agents tested included chloramphenicol, tetracycline, cefoxitin, ceftazidime, imipenem, and trimethoprim–sulfamethoxazole. Results were interpreted using CLSI breakpoints.

### Whole-genome sequencing

Genomic DNA was extracted using the Qiagen DNeasy Blood & Tissue Kit (QIAGEN, Hilden, Germany) following the manufacturer’s instructions. Sequencing libraries were prepared with the Nextera XT DNA Library Preparation Kit (Illumina, San Diego, CA, USA) and sequenced on an Illumina MiSeq platform, generating paired-end reads (2 × 250 bp). Raw read quality was assessed using FastQC v0.11.9.

### Genome assembly and annotation

De novo genome assembly was performed using Unicycler v0.5.1 in normal mode. Assembly quality metrics were evaluated with QUAST v5.0.2. Genome completeness was assessed using BUSCO v6.0.0 against the Vibrionales_odb10 dataset, utilizing Prodigal v2.6.3 for gene prediction. Automated genome annotation was conducted using Bakta v1.8.2. Circular chromosome maps were generated using Proksee for visualization and contextual interpretation of genomic features.

### Multi-locus sequence typing (MLST)

Multi-locus sequence typing was performed using the MLST tool v2.23.0 to assign sequence type (ST) based on the V. cholerae MLST scheme.

### Identification of antimicrobial resistance determinants

Antimicrobial resistance determinants were identified using the Resistance Gene Identifier (RGI) v6.0.3 with the Comprehensive Antibiotic Resistance Database (CARD). Analyses were performed using the “Perfect” and “Strict” detection paradigms. In CARD-RGI, a “Perfect” hit corresponds to an exact match to a curated reference resistance determinant or curated resistance-associated variant, representing the highest-confidence and most specific category. A “Strict” hit corresponds to a non-identical but high-confidence match that exceeds the curated model-specific similarity/bit-score threshold, and, where applicable, includes secondary screening for key resistance-associated mutations. Accordingly, the “Perfect” model is more conservative and maximizes specificity, whereas the “Strict” model increases sensitivity for the detection of previously described variants or closely related homologs while maintaining curated confidence thresholds. Loose hits were not considered in this study in order to avoid overinterpretation of low-confidence predictions [14].

### Virulence factor analysis

Virulence-associated genes were identified using ABRicate v1.0.1 with the Virulence Factor Database (VFDB, version 2023). Thresholds of ≥ 90% nucleotide identity and ≥ 40% coverage were applied. The coverage threshold was reduced from the default to minimize false negatives, particularly for genes located at the edges of contigs—a common artifact in short-read sequencing assemblies. Identified genes were subsequently grouped into functional categories, including adhesion and colonization, toxins, secretion systems, quorum sensing, iron acquisition, and antimicrobial peptide resistance.

### SNP-based phylogenetic analysis and comparative genomics

For comparative genomic and phylogenetic analyses, 62 publicly available Vibrio cholerae genomes were retrieved from the NCBI database (Table 1) and analyzed together with the study isolate V86/24, yielding a total dataset of 63 genomes (*N* = 63). Genome selection was based on epidemiological, temporal, and phylogenetic criteria, prioritizing isolates representative of the ST170/L3b.2 lineage circulating in the Americas. Crucially, to provide a robust evolutionary framework, we included representative genomes from sister clades within the broader L3b lineage—specifically ST75 isolates from Russia and China representing sublineages L3b.1, L3b.3, and L3b.4. This approach was necessary to accurately resolve the phylogenetic positioning of V86/24 within the global non-pandemic O1 population. A limited number of reference pandemic (7PET) strains were also included for outgroup context.The dataset included isolates from human and environmental sources collected across different years and geographic regions, allowing assessment of lineage stability, regional clustering, and divergence patterns.

**Table 1:** Epidemiological and genomic data and pathogenicity island profile of public *Vibrio cholerae* O1 isolates included in the comparative dataset (*n* = 62) used together with the study isolate V86/24 (total *N* = 63)

Strain	Accession	Country	Year	Source	ST	Lineage	Lineage in Domman et al. Science, 2017	Vibrio cholerae pathogenic island			
								VPI-1	VPI-2	VSP-1	VSP-2
85	GCA_002196255.1	Ukraine	NA	Human	75	L3b (L3b.3)		+	+	-	-
124	GCA_003057085.1	Russia	2015	Environmental	75	L3b (L3b.3)		-	-	-	-
391	ERR163261	Mexico	2010	Environmental	170	L3b (L3b.2)	MX-2	+	+	-	-
586	ERR163262	Mexico	2010	Environmental	170	L3b (L3b.2)	MX-2	+	+	-	-
667	ERR163264	Mexico	2010	Environmental	170	L3b (L3b.2)	MX-2	+	+	-	-
819	ERR163265	Mexico	2010	Environmental	170	L3b (L3b.2)	MX-2	+	+	-	-
866	GCA_002204085.1	Ukraine	1996	Environmental	75	L3b (L3b.2)		-	-	-	-
1127	ERR108519	Mexico	1999	Human	170	L3b (L3b.2)	MX-2	+	+	-	-
1876	ERR108520	Mexico	1999	Human	170	L3b (L3b.2)	MX-2	+	+	-	-
2283	ERR163266	Mexico	2010	Environmental	170	L3b (L3b.2)	MX-2	+	+	-	-
2284	ERR163267	Mexico	2010	Environmental	170	L3b (L3b.2)	MX-2	+	+	-	-
2687	GCA_003057075.1	Russia	2015	Environmental	75	L3b (L3b.3)		-	-	-	-
2613	GCA_003057055.1	Russia	2015	Environmental	75	L3b (L3b.3)		+	-	-	-
2688	GCA_003056975.1	Russia	2015	Environmental	75	L3b (L3b.3)		+	-	-	-
00/3079	ERR2008415	Mexico	2000	Human	170	L3b (L3b.2)	MX-2	+	+	-	-
00/3275	ERR2008745	Mexico	2000	Human	170	L3b (L3b.2)	MX-2	+	+	-	-
03 − 1_S94	QZRO00000000	China	2003	Human	169	L3b (L3b.2)		+	+	-	-
06–11_S39	QZRW00000000	China	2006	Human	75	L3b (L3b.4)		+	+	+	-
07–16_S121	QZSC00000000	China	2007	Human	75	L3b (L3b.4)		+	+	+	-
07 − 3_S124	QZSF00000000	China	2007	Human	75	L3b (L3b.4)		+	-	+	-
07 − 4_S125	QZSG00000000	China	2007	Human	75	L3b (L3b.4)		-	-	+	-
08–12_S128	QZSI00000000	China	2008	Environmental	75	L3b (L3b.4)		-	+	+	-
08–14_S129	QZSJ00000000	China	2008	Environmental	75	L3b (L3b.4)		+	-	+	-
08–19_S131	QZSM00000000	China	2008	Human	75	L3b (L3b.4)		-	+	+	-
08–20_S133	QZSN00000000	China	2008	Human	75	L3b (L3b.4)		-	-	+	-
08–21_S134	QZSO00000000	China	2008	Human	75	L3b (L3b.4)		-	+	+	-
11 − 2_S78	QZUH00000000	China	2011	Human	182	L3b (L3b.1)		+	+	-	-
11 − 3_S23	QZUI00000000	China	2011	Human	170	L3b (L3b.2)		+	+	-	-
33224_2_213	ERS3907678	China	2021	Human	170	L3b.2		+	+	-	-
33224_2_224	ERR5312518	Yemen	2021	Human	170	L3b.2		+	+	-	-
56_2	GCA_002204105.1	Ukraine	1995	Human	75	L3b (L3b.3)		-	-	-	-
99/1127	ERR2008354	Mexico	1999	Human	170	L3b (L3b.2)	MX-2	+	+	-	-
99/2110	ERR2008356	Mexico	1999	Human	170	L3b (L3b.2)	MX-2	+	-	-	-
99/2118	ERR2008431	Mexico	1999	Human	170	L3b (L3b.2)	MX-2	+	+	-	-
2012Env-9	GCA_000788715.2	Haiti	2012	Environmental	171	L9 (L9.3)		-	-	-	-
Env-390	GCA_001854425.1	Haiti	2013	Environmental	171	L9 (L9.3)		-	-	-	-
I-1471	GCA_000818865.1	Russia	2011	Environmental	171	L3b (L3b.1)		+	+	-	-
M139	GCA_001637545.1	Turkmenistan	1965	Environmental	171	L3b (L3b.1)		+	-	-	-
M1395	GCA_001515105.1	Russia	1981	Environmental	75	L3b (L3b.1)		+	-	-	-
M1399	GCA_001515085.1	Russia	1982	Environmental	75	L3b (L3b.1)		+	-	-	-
M1501	GCA_001637575.1	Russia	2011	Human	75	L3b (L3b.1)		+	-	-	-
M1518	GCA_001641685.1	Russia	2012	Environmental	75	L3b (L3b.3)		+	-	-	-
M1524	GCA_001641705.1	Russia	2013	Environmental	75	L3b (L3b.3)		+	-	-	-
M299	GCA_001637555.1	Turkmenistan	1965	Human	75	L3b (L3b.2)		-	+	-	-
P-18,778	GCA_002196065.1	Russia	2005	Human	75	L3b (L3b.1)		+	-	-	-
P-18,785	GCA_000338215.2	Russia	2005	Human	75	L3b (L3b.1)		+	-	-	-
TUC_VC182_14	ERR1024531	Argentina	2014	Human	170	L3b.2	MX-2	+	+	-	-
TUC_VC849_07	ERR1024528	Argentina	2007	Human	170	L3b.2	MX-2	+	+	-	-
V1/91	SRR31308668	Brazil	1991	Human	69	L2	7PET	+	+	+	+
V1077/92	SRR31308661	Brazil	1992	Human	69	L2	7PET	+	+	+	+
V11/91	SRR31308667	Brazil	1991	Human	69	L2	7PET	+	+	+	+
V15/91	SRR31308667	Brazil	1991	Human	69	L2	7PET	+	+	+	+
V14671/95	SRR29412334	Brazil	1995	Human	69	L2	7PET	+	+	+	+
V292/91	SRR31308665	Bolivia	1991	Human	69	L2	7PET	+	+	+	+
V38/91	SRR31308666	Brazil	1991	Human	69	L2	7PET	+	+	+	+
V400/91	SRR31308664	Brazil	1991	Human	69	L2	7PET	+	+	+	+
V430/91	SRR31308663	Brazil	1991	Human	69	L2	7PET	+	+	+	+
V603/91	SRR31308662	Brazil	1991	Human	69	L2	7PET	+	+	+	+
V86/24	SRR36916814	Brazil	2024	Human	170	L3b.2	MX-2	+	+	-	-
VC_2283_05	ERR353353	Argentina	2005	Human	170	L3b.2	MX-2	+	+	-	-
VC_521_09	ERR353352	Paraguay	2009	Human	170	L3b.2	MX-2	+	+	-	-
ZJ11010	SRR2738126	China	2011	Human	170	L3b.2		+	+	-	-

*NA = not available

The lineage classification follows the nomenclature proposed by Domman et al. (Science, 2017) for non-7PET Vibrio cholerae lineages. The designation "L3b (L3b.2)" indicates that the isolate belongs to clade L3b, subclade L3b.2. The nomenclature "MX-2" refers to a sublineage geographically associated with isolates from Mexico and other countries in the Americas, as described in the same study

Reads and/or assembled genomes were mapped using minimap2, followed by processing with samtools and variant calling with bcftools. Recombination was detected and masked using Gubbins, and the resulting recombination-free core genome alignment was used for downstream phylogenetic inference. Core-genome single nucleotide polymorphisms (SNPs) were used to infer a maximum-likelihood phylogeny with IQ-TREE. Phylogenetic trees and associated metadata were visualized using iTOL.

### Pangenome analysis

Pangenome analysis was conducted using Panaroo v1.3.7 in strict mode, following annotation with Bakta v1.8.2. Panaroo generated gene presence/absence matrices and a core genome alignment by retaining gene clusters present in ≥ 95% of genomes (core-alignment threshold), with contamination-aware clustering and MAFFT v7.526 for alignment. Separately, for descriptive purposes, gene clusters were assigned to frequency classes based on their prevalence across genomes: core (≥ 99%), soft-core (95–<99%), shell (15–<95%), and cloud (< 15%). A maximum-likelihood phylogeny was inferred from the Panaroo core alignment using IQ-TREE v2 with ModelFinder, supported by 1,000 ultrafast bootstrap and 1,000 SH-aLRT replicates. The accessory genome was visualized via hierarchical clustering of Jaccard distances, and V86/24-exclusive clusters were mapped to their genomic context.

## Results

Isolate V86/24 was identified as *Vibrio cholerae* O1 Ogawa and exhibited a predominantly susceptible antimicrobial profile to chloramphenicol, tetracycline, cefoxitin, ceftazidime, and trimethoprim–sulfamethoxazole except for an intermediate to imipenem.

The final assembly consisted of 82 contigs with a total predicted genome size of approximately 3.97 Mb. The largest contig measured 486,240 bp, while the assembly contiguity metrics were an N50 of 197,303 bp and an N90 of 55,579 bp.

Whole-genome sequencing and in silico typing classified isolate V86/24 as sequence type ST170, belonging to lineage L3b.2 and sublineage MX-2. Analysis of pathogenicity islands revealed the presence of VPI-1 and VPI-2, whereas the seventh pandemic-associated islands VSP-1 and VSP-2 were absent, supporting its classification within a non-7PET lineage (Table 1).

Resistome analysis identified the presence of *var*G, a gene encoding a metallo-β-lactamase associated with the var regulon and considered part of the intrinsic genomic repertoire of *V. cholerae*. In addition, components of the *alm* gene set, which are linked to lipopolysaccharide modification, were detected, together with regulatory determinants potentially associated with subtle modulation of antimicrobial susceptibility. No acquired resistance genes were identified, in agreement with the largely susceptible phenotypic profile observed.

Screening for virulence-associated genes revealed the absence of classical CTXΦ-associated toxin genes, including *ctx*A, *ctx*B, *ace*, and *zot*, confirming a non-toxigenic genomic profile. Conversely, multiple determinants associated with intestinal colonization, persistence, and alternative virulence were detected. These included the accessory colonization factors *acf*A–*acf*D, outer membrane protein *omp*U, and a complete toxin-coregulated pilus (TCP) locus together with its regulatory components. Additional virulence-associated features comprised hemolysin (*hly*A), a complete RTX/MARTX toxin cluster (*rtx*A–*rtx*E), and an extensive repertoire of type VI secretion system (T6SS) genes encompassing structural, regulatory, and effector-associated components. Genes involved in quorum sensing were also identified, indicating the presence of regulatory networks associated with population-level behavior and environmental adaptation.

**Fig. 1:**
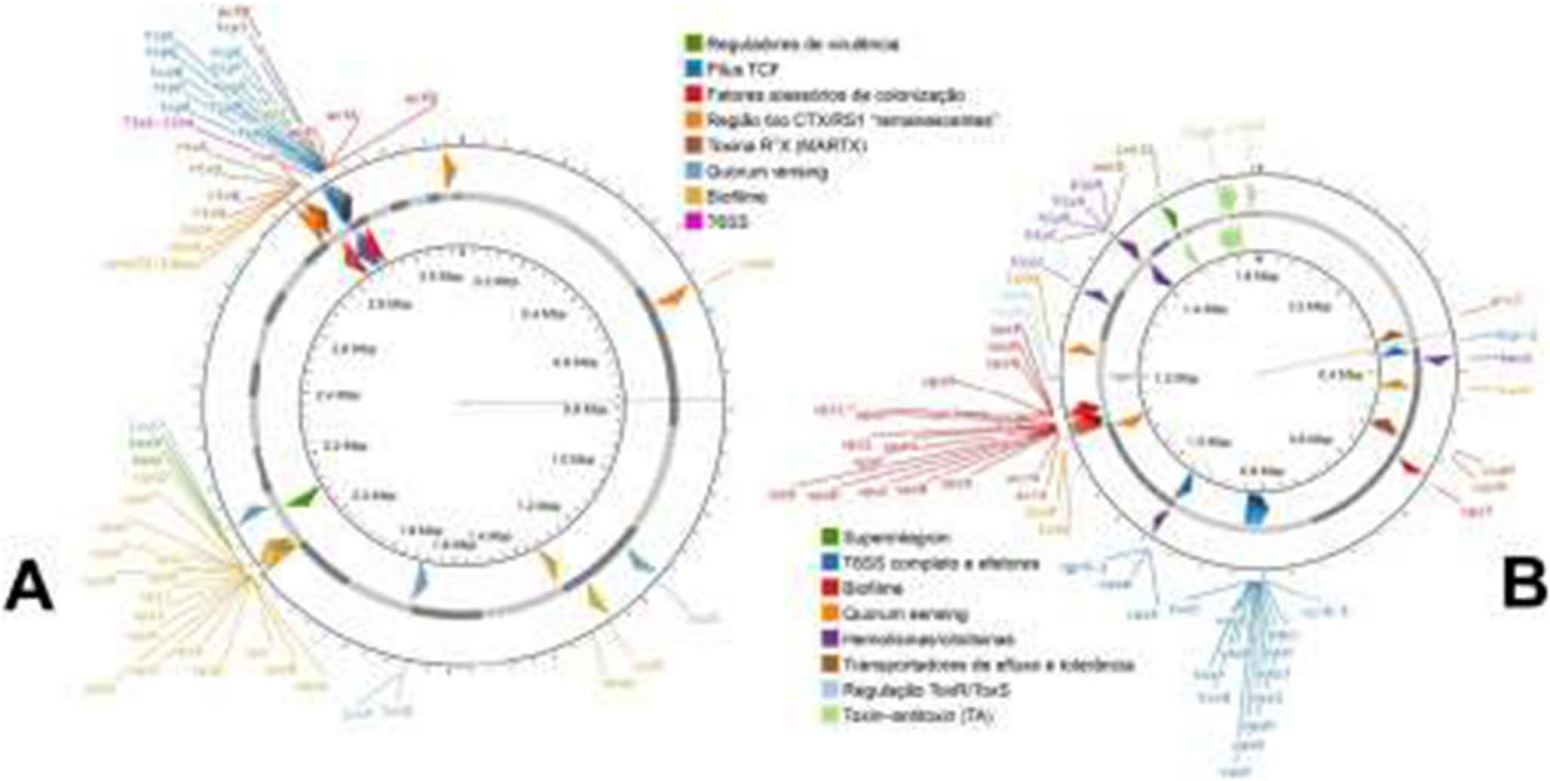
Circular mapping of major virulence and adaptation determinants in the bipartite genome of Vibrio cholerae V86/24. (A) Chromosome 1 (~ 3.0 Mb), highlighting gene clusters associated with colonization (ACF accessory factors in red; TCP pilus in blue), RTX family toxins (brown), and global virulence regulators (toxR/S). Notably, remnant CTX/RS1-like regions are present, consistent with the absence of a fully functional CTXΦ prophage. (B) Chromosome 2 (~ 1.6 Mb), showing high density of superintegron genes (green), a complete type VI secretion system cluster (T6SS; blue), and biofilm-essential genes (vps operons; red). External annotations indicate gene location and transcription direction (arrows), colored according to functional categories

The genome of isolate V86/24 consisted of two chromosomes with distinct functional enrichment. Chromosome 1 (~ 3.15 Mb) harbored major loci related to intestinal colonization and virulence regulation, including the *tox*R/*tox*S regulatory axis, TCP and ACF gene clusters, and the RTX/MARTX toxin region. Chromosome 2 (~ 1.61 Mb) was enriched in determinants linked to environmental persistence and bacterial competition, including a broad and apparently complete T6SS, quorum sensing systems, and extensive biofilm-associated operons (Fig. 1).

**Fig. 2:**
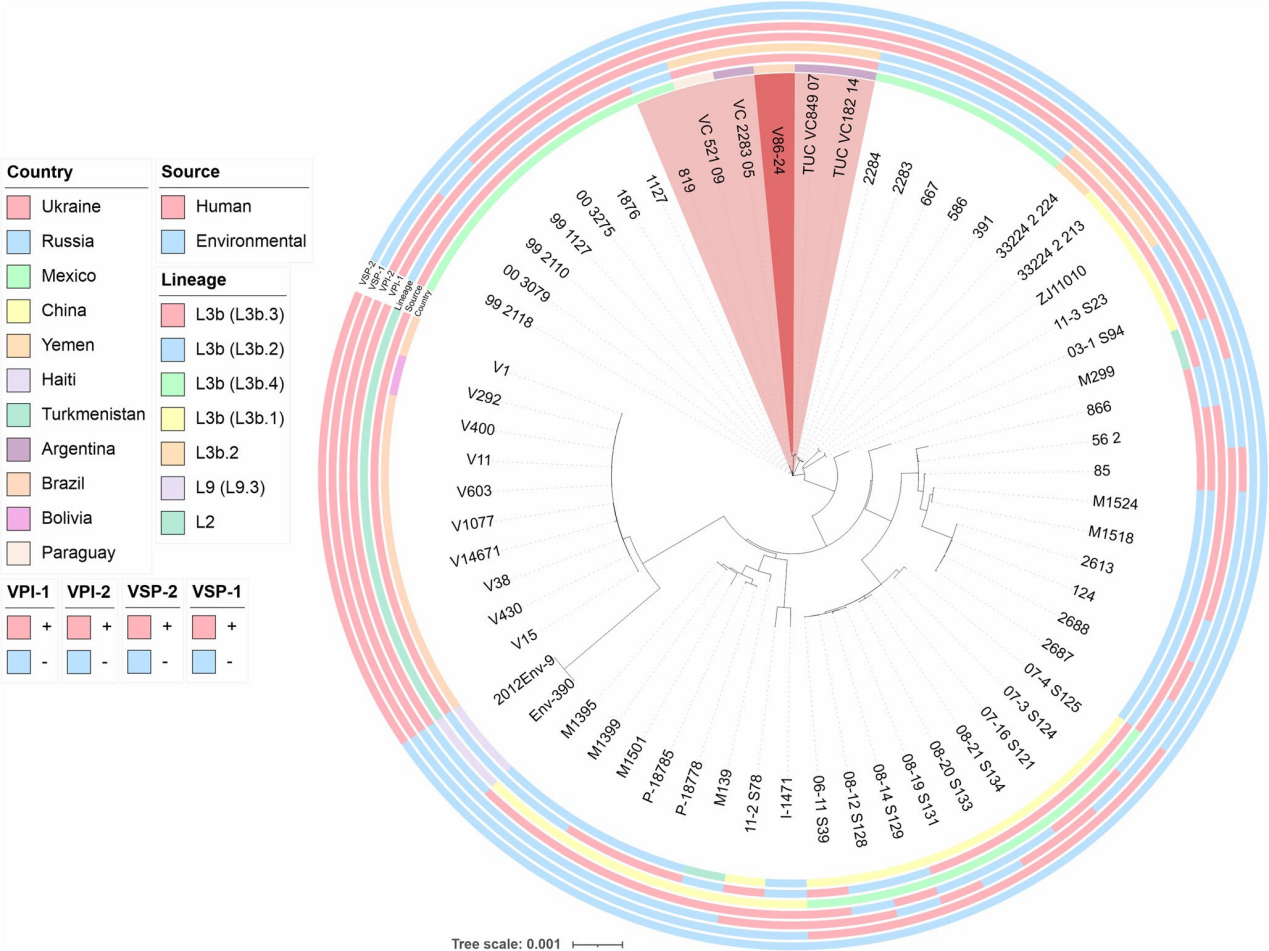
Maximum-likelihood SNP phylogeny of Vibrio cholerae based on core-genome SNPs. The phylogeny was inferred with IQ-TREE from a SNP alignment obtained by mapping against the reference genome V. cholerae O1 biovar El Tor strain N16961 (chromosome I: AE003852.1; chromosome II: AE003853.1). Strain V86/24 (highlighted in red) falls within the ST170 clade. The heatmap indicates geographic origin, isolation source, and presence/absence of pathogenicity islands (VPI/VSP)

For comparative genomic analyses, the study isolate V86/24 was analyzed together with 62 publicly available V. cholerae genomes (total *N* = 63), including the Haitian non-toxigenic environmental O1 strains 2012Env-9 and 2012Env-390. In the SNP-based maximum-likelihood phylogeny, isolate V86/24 clustered within the ST170/L3b.2/MX-2 clade, grouping closely with isolates from Mexico, Argentina, and Paraguay collected between 1999 and 2014 from both human and environmental sources (Fig. 2). In contrast, the Haitian strains 2012Env-9 and 2012Env-390 did not cluster closely with V86/24 and occupied a separate branch, indicating that the Brazilian isolate belongs to a distinct non-7PET American background. Pandemic 7PET strains formed a distinct and clearly separated cluster.

Pangenome analysis further supported the close relatedness of V86/24 to the ST170 background while highlighting accessory-genome microvariation consistent with mobile genetic elements. In the full dataset, the pangenome comprised 4,485 gene clusters, including a conserved core of 691 clusters and a substantial accessory fraction (shell and cloud), indicating broad gene-content diversity across non-7PET lineages. Focusing on V86/24, eight gene clusters were uniquely present in this isolate. Seven of these unique clusters mapped to a single region on contig_5 (approximately 204–220 kb), overlapping a phage-like module that includes a terminase large subunit and additional phage-associated proteins, consistent with a prophage-derived acquisition. The remaining unique cluster corresponded to *rst*C on contig_20, a gene commonly associated with RS/CTXΦ-related regions, supporting the presence of CTX/RS remnant signatures despite the absence of *ctx*AB, *ace*, and *zot*. Accordingly, core-alignment genes (≥ 95% prevalence) and the strict core class (≥ 99% prevalence) represent complementary summaries of shared gene content.

Accessory-genome clustering based on Panaroo presence/absence profiles recapitulated the core-genome grouping of V86-24, which clustered with TUC_VC182_14 and TUC_VC849_07 (Argentina). Within this cluster, accessory variation was largely restricted to sparse, low-frequency gene blocks rather than broad lineage-defining acquisitions (Fig. S2).

## Discussion

The present study reports the genomic characterization of a *Vibrio cholerae* O1 Ogawa isolate recovered from a human case in Brazil in 2024 in the absence of travel history, providing evidence for the circulation of non-pandemic O1 lineages outside classical endemic contexts. Although toxigenic O1 strains remain the primary drivers of epidemic cholera, this finding reinforces that non-toxigenic O1 isolates continue to be epidemiologically relevant and may contribute to sporadic human disease [15, 16].

The absence of CTXΦ-associated toxin genes (*ctx*AB, *ace*, and *zot*) in isolate V86/24 strongly suggests a low likelihood of classical cholera. However, increasing evidence indicates that the pathogenic potential of *V. cholerae* cannot be inferred solely from toxigenicity. Non-toxigenic isolates may cause gastroenteritis through alternative mechanisms, particularly when they retain efficient colonization capacity and harbor factors associated with cytotoxicity and microbial competition [15, 16]. In this context, the genomic profile of V86/24 supports a pathogenic architecture centered on intestinal fitness rather than toxin-mediated disease.

A key feature of isolate V86/24 is the presence of a complete colonization and regulatory framework, including the TCP/ACF axis and the *tox*R/*tox*S–*tox*T regulatory network. These elements provide biological possibility for intestinal establishment even in the absence of cholera toxin and are consistent with previous observations that TCP-positive, *ctx*-negative strains can be associated with symptomatic infection [16, 17]. The retention of these systems, together with a fully assembled RTX/MARTX cluster, suggests that cytotoxic effects and host–pathogen interactions may contribute to disease manifestations distinct from classical cholera [15, 16].

Beyond host interaction, the extensive repertoire of type VI secretion system (T6SS) components, quorum sensing circuits, and biofilm-associated genes highlights a genome optimized for environmental persistence and bacterial competition. Such features are increasingly recognized as critical determinants of *V. cholerae* ecology, facilitating survival in aquatic reservoirs, surface attachment, and interaction with complex microbial communities [18, 19]. This ecological versatility provides a plausible explanation for how non-pandemic lineages may persist regionally and intermittently give rise to human exposure events without overt epidemic spread [15, 19].

From an evolutionary aspect, the combination of VPI-1 and VPI-2 with the absence of VSP-1 and VSP-2 places isolate V86/24 outside the seventh-pandemic lineage framework, aligning it with the ST170/L3b.2/MX-2 background identified in our comparative dataset and, more broadly, with non-7PET O1 lineages related to the L3b framework previously associated with regional circulation and sporadic human infection. The SNP-based phylogenetic clustering with historical isolates from Mexico, Argentina, and Paraguay supports a scenario of long-term regional circulation rather than recent introduction of a pandemic clone. These findings emphasize that non-7PET O1 lineages may represent stable, geographically structured populations with sustained capacity for human infection [20, 21].

The inclusion of the Haitian non-toxigenic environmental O1 strains 2012Env-9 and 2012Env-390 provides additional context for interpreting V86/24. Azarian et al. showed that these ctxAB-negative O1 strains represent environmentally persistent non-toxigenic backgrounds of epidemiological relevance. In our phylogenetic reconstruction, however, these Haitian isolates did not cluster closely with V86/24, indicating that the Brazilian isolate does not derive from the same immediate branch. Instead, V86/24 falls within an ST170/L3b.2/MX-2 American lineage, supporting the view that clinically relevant non-toxigenic O1 strains may arise from multiple distinct evolutionary backgrounds rather than from a single non-toxigenic O1 lineage [8].

Furthermore, the absence of clear evidence for the canonical WASA-1 island is noteworthy, as WASA-1 has been described as a characteristic prophage/genomic island associated with the West African–South American lineage. In V86/24, inspection of the pepN locus did not support the presence of the canonical WASA-1 integration pattern, and the prophage-like module identified elsewhere in the genome could not be confidently assigned to this island. These findings suggest that, despite retaining phage-associated accessory content and genomic plasticity, V86/24 does not show the specific WASA-1 signature classically linked to the WASA background, which is consistent with its placement within a distinct non-7PET O1 lineage rather than within the classical WASA framework [22].

Pangenome analysis using Panaroo provides additional resolution to contextualize V86/24 within non-7PET O1 diversity. Across the comparative dataset, the pangenome comprised 4,485 gene clusters, including a conserved core (691 clusters) and a substantial accessory fraction (shell and cloud), consistent with extensive gene-content variation driven by mobile elements and niche adaptation in non-pandemic lineages [23–25]. Here, the core (691 clusters) refers to the strict core, defined as gene clusters present in ≥ 99% of genomes. Importantly, V86/24 retains the core and most of the soft-core repertoire of its closest phylogenomic neighbors, supporting a stable backbone in which differences are largely concentrated in the accessory genome. The relatively small strict core is expected given the inclusion of multiple O1 lineages spanning non-7PET and reference 7PET genomes, which increases gene-content heterogeneity and reduces the number of clusters shared by nearly all isolates.

Focusing on V86/24, eight gene clusters were uniquely detected in this isolate and mapped primarily to a single region on contig_5 (approximately 204–220 kb), overlapping a phage-like module that includes a terminase large subunit and other phage-associated proteins, consistent with a prophage-derived acquisition. Such localized gains in the mobilome are consistent with the fine-scale accessory variation observed among environmentally persistent O1 lineages and may modulate ecological fitness and competition without implying seventh-pandemic ancestry [19, 26]. In parallel, a unique cluster corresponding to *rstC* was detected on contig_20, providing a specific marker compatible with CTX/RS-associated remnant structure.

The concordance between core-genome phylogeny and accessory-genome clustering, together with the limited and patchy nature of accessory differences, is consistent with microevolution driven by the mobilome rather than large-scale acquisition of seventh-pandemic–associated islands (Fig. S2).

The antimicrobial susceptibility profile of V86/24 further supports its classification as a non-pandemic lineage, showing broad susceptibility with only intermediate response to imipenem. The detection of *var*G, an intrinsic metallo-β-lactamase gene within the var regulon, provides a plausible genomic background for subtle modulation of β-lactam susceptibility without conferring overt resistance. This observation underscores the importance of interpreting antimicrobial phenotypes in *V. cholerae* within a framework that considers intrinsic resistance determinants and regulatory effects, rather than focusing exclusively on acquired resistance genes [16, 19].

An additional aspect of epidemiological relevance is the identification of CTX/RS1-like remnant regions in the genome of V86/24, in the absence of a functional CTXΦ prophage. Such remnants indicate historical or partial exposure to mobile genetic elements associated with toxigenic lineages and highlight the inherent genomic plasticity of *V. cholerae* [27, 28]. In environments where toxigenic strains and CTXΦ phages circulate, non-toxigenic O1 isolates retaining colonization and regulatory competence may serve as receptive backgrounds for future acquisition of toxigenicity determinants, potentially altering their public health [15, 27–29].

In summary, these findings support the interpretation of isolate V86/24 as a non-toxigenic *Vibrio cholerae* O1 lineage with a genomic architecture favoring colonization, competition, and environmental persistence rather than epidemic cholera [15, 19]. Importantly, this study represents the outcome of the first official genomic investigation conducted within the Brazilian national epidemiological surveillance framework, involving the reference laboratory for bacterial enteric infections, during the transition from pulsed-field gel electrophoresis–based surveillance (PulseNet Americas) to whole-genome sequencing for routine characterization and epidemiological investigation. From a surveillance perspective, such isolates should not be dismissed as clinically irrelevant; rather, they function as sensitive sentinels of *V. cholerae* circulation across aquatic and human-associated environments and exemplify the added resolution provided by genome-based surveillance strategies that extend beyond the detection of *ctx*AB alone [15, 21].

To our knowledge, this study represents a rare genomic characterization of a non-toxigenic Vibrio cholerae O1 isolate from an autochthonous human case in Brazil after an 18-year absence of reported cases, and provides relevant evidence for genomic surveillance of clinically significant non-7PET O1 lineages.

## Conclusion

This study describes a non-toxigenic Vibrio cholerae O1 Ogawa isolate associated with a sporadic human case in Brazil, whose genomic profile is consistent with a non-pandemic ST170/L3b.2/MX-2 lineage. Despite the absence of CTXΦ-associated toxin genes, the presence of conserved colonization and persistence determinants supports the capacity of such lineages to cause human infection outside endemic or outbreak settings. The phylogenetic placement of this isolate within a regionally circulating lineage in the Americas, together with signatures of genomic plasticity, highlights the importance of genomic surveillance beyond classical toxigenicity markers. Non-toxigenic *V. cholerae* O1 strains should therefore be regarded as sentinels of ongoing circulation and potential evolutionary risk, rather than as epidemiological dead ends.

## Supplementary Information

Below is the link to the electronic supplementary material.

Supplementary Material 1. Supplementary Figure S1. Linear representation of contig_5 of V86/24 highlighting the prophage-like module (approximately 191,917-224,269 bp) and the positions of V86/24-exclusive gene clusters mapped by Panaroo (locus tags KAFEAP_01501, KAFEAP_01508, KAFEAP_01517, KAFEAP_01519, KAFEAP_01524, KAFEAP_01528, and KAFEAP_01531).

Supplementary Material 2. Supplementary Figure S2. Accessory-genome presence/absence clustering from the Panaroo pangenome. The left panel shows hierarchical clustering of genomes based on accessory gene content, and the right panel displays the accessory gene presence/absence matrix (blue, present; light/grey, absent). V86/24 is highlighted (red box) and clusters with its closest relatives (TUC_VC182_14 and TUC_VC849_07), indicating a broadly shared accessory repertoire within this group, with differences concentrated in a limited number of low-frequency accessory loci.

## Data Availability

Raw sequencing reads for isolate V86/24 are available in the NCBI Sequence Read Archive (SRA) under accession SRR36916814. Additional data supporting the findings of this study are included in the article.
