# Supplementary figures and images for: Genomic and epidemiological insights into a non-toxigenic Vibrio cholerae O1 Ogawa from an autochthonous case in Brazil
Source: Eur J Clin Microbiol Infect Dis. 2026 Apr 15;45(8):2291–301. doi: 10.1007/s10096-026-05495-4 (PMC13428693; doi:10.1007/s10096-026-05495-4)

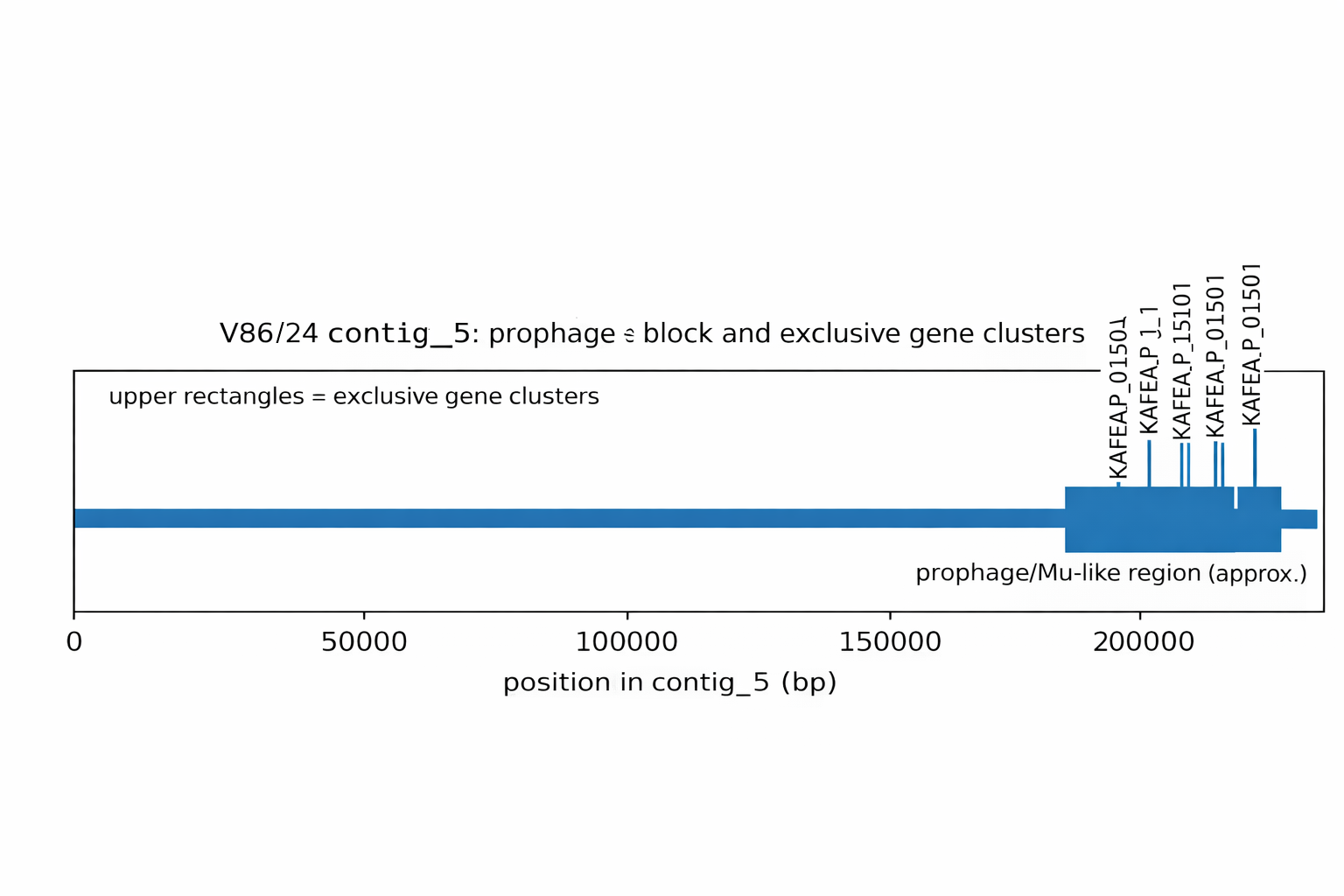

Supplement: Supplementary file 1 — Supplementary Material 1. Supplementary Figure S1. Linear representation of contig_5 of V86/24 highlighting the prophage-like module (approximately 191,917-224,269 bp) and the positions of V86/24-exclusive gene clusters mapped by Panaroo (locus tags KAFEAP_01501, KAFEAP_01508, KAFEAP_01517, KAFEAP_01519, KAFEAP_01524, KAFEAP_01528, and KAFEAP_01531). [file 10096_2026_5495_MOESM1_ESM.png]

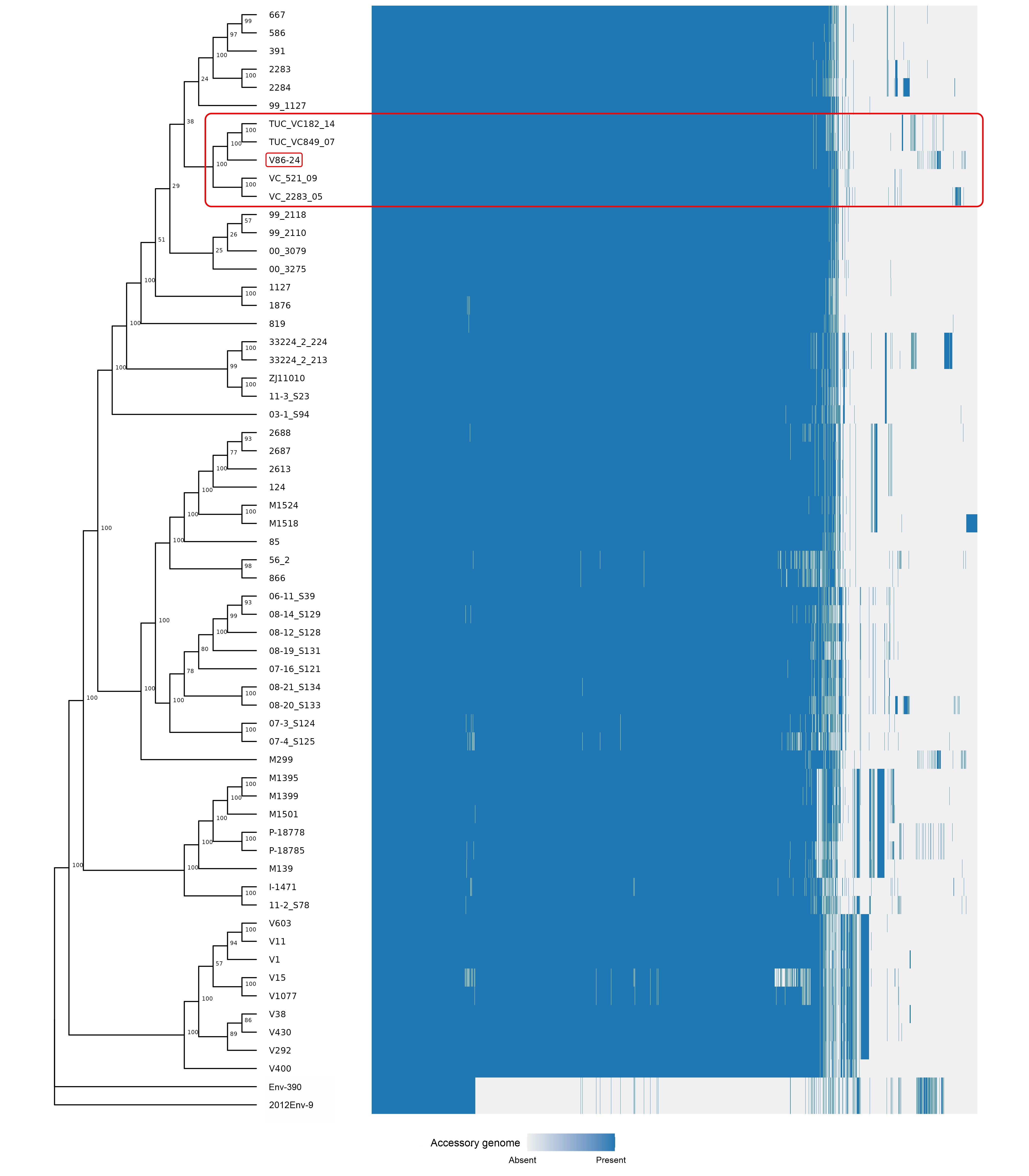

Supplement: Supplementary file 2 — Supplementary Material 2. Supplementary Figure S2. Accessory-genome presence/absence clustering from the Panaroo pangenome. The left panel shows hierarchical clustering of genomes based on accessory gene content, and the right panel displays the accessory gene presence/absence matrix (blue, present; light/grey, absent). V86/24 is highlighted (red box) and clusters with its closest relatives (TUC_VC182_14 and TUC_VC849_07), indicating a broadly shared accessory repertoire within this group, with differences concentrated in a limited number of low-frequency accessory loci. [file 10096_2026_5495_MOESM2_ESM.jpg]
